# Single-voxel delay map from long-axial field-of-view PET scans

**DOI:** 10.3389/fnume.2024.1360326

**Published:** 2024-04-19

**Authors:** Frederik Bay Nielsen, Ulrich Lindberg, Heloisa N. Bordallo, Camilla Bardram Johnbeck, Ian Law, Barbara Malene Fischer, Flemming Littrup Andersen, Thomas Lund Andersen

**Affiliations:** ^1^ Department of Clinical Physiology & Nuclear Medicine, Copenhagen University Hospital—Rigshospitalet, Copenhagen, Denmark; ^2^ Faculty of Natural and Life Sciences, Niels Bohr Institute, University of Copenhagen, Copenhagen, Denmark; ^3^ Department of Clinical Medicine, Faculty of Health and Medical Sciences, University of Copenhagen, Copenhagen, Denmark

**Keywords:** dynamic whole-body PET, kinetic modeling, one-tissue compartmental modeling, delay correction, delay map

## Abstract

**Objective:**

We present an algorithm to estimate the delay between a tissue time-activity curve and a blood input curve at a single-voxel level tested on whole-body data from a long-axial field-of-view scanner with tracers of different noise characteristics.

**Methods:**

Whole-body scans of 15 patients divided equally among three tracers, namely [^15^O]H_2_O, [^18^F]FDG and [^64^Cu]Cu-DOTATATE, which were used in development and testing of the algorithm. Delay times were estimated by fitting the cumulatively summed input function and tissue time-activity curve with special considerations for noise. To evaluate the performance of the algorithm, it was compared against two other algorithms also commonly applied in delay estimation: name cross-correlation and a one-tissue compartment model with incorporated delay. All algorithms were tested on both synthetic time-activity curves produced with the one-tissue compartment model with increasing levels of noise and delays between the tissue activity curve and the blood input curve. Whole-body delay maps were also calculated for each of the three tracers with data acquired on a long-axial field-of-view scanner with high time resolution.

**Results:**

Our proposed model performs better for low signal-to-noise ratio time-activity curves compared to both cross-correlation and the one-tissue compartment models for non-[^15^O]H_2_O tracers. Testing on synthetically produced time-activity curves showed only a small and even residual delay, while the one-tissue compartment model with included delay showed varying residual delays.

**Conclusion:**

The algorithm is robust to noise and proves applicable on a range of tracers as tested on [^15^O]H_2_O, [^18^F]FDG and [^64^Cu]Cu-DOTATATE, and hence is a viable option offering the ability for delay correction across various organs and tracers in use with kinetic modeling.

## 1 Introduction

Dynamic positron emission tomography (PET) scans are widely used as a non-invasive technique to estimate physiological parameters of different tissues, such as the blood perfusion of organs using [^15^O]H_2_O (1–3) or the metabolism of glucose in the brain or tumors using [^18^F]FDG (4). Kinetic parameters of interest can be calculated by modeling the tissue time-activity curve (TAC) as a response to an input curve or input function, typically employed in the form of compartmental models (5, 6).

Prior to kinetic modeling and particularly for the short-lived isotopes, it is essential to correct for relative transport delay between the input function, i.e., the tracer concentration in the blood as a function of time, and the tissue response curve, which are typically acquired from two different anatomical locations in the body. On a clinical standard size PET scanner of approximately 25 cm and if the organ of interest cannot be in the same field of view as a large arterial space, the input function is usually measured by sampling blood from an arterial cannulation—also called an arterial input function (AIF)—while on whole-body scanners the input function is often derived from a segmentation of the aorta—called an image-derived input function (IDIF)—that does not require any invasive measurements (7, 8).

Long-axial field-of-view (LAFOV) PET scanners are capable of capturing much more of the available signal due to the extended coverage, enabling dynamic scans with faster frame rates of around one second, reducing motion artifacts and increasing the accuracy of kinetic modeling and generation of parametric images (9–11). The increased sensitivity of these scanners also allows for lowering the dose of radiotracer administered to the patient, ultimately lessening the radiation exposure experienced by the patient (12). In addition, the high spatial resolution of LAFOV scanners reduces partial volume effects and allows for a more accurate image-derived input function (7, 8, 13).

Modeling the kinetics and physiological parameters of an organ often involves using a single mean TAC for the entire organ. This approach improves the signal-to-noise ratio and the robustness of the subsequent fit. However, this method has a drawback as it potentially yields less accurate regional modeling results by neglecting the heterogeneity within the organ (14). To address this issue and analyze the variability of kinetic parameters across different regions of the organ, single-voxel modeling can be employed. Despite its advantages, single-voxel modeling faces significant challenges, primarily due to the low signal-to-noise ratio. The advancement of LAFOV scanners, which benefit from an axial FOV of 1 m or more, has mitigated this problem. These scanners offer enhanced sensitivity and improved time resolution, making the kinetic modeling of single voxels more reliable and accurate (15).

Another aspect to consider is the kinetic modeling of organs where regions differ significantly in terms of delay time between the input function and the tissue curve. For instance, in the case of the brain in patients with single-sided carotid stenosis, employing an average delay based on the mean organ TAC may lead to inaccuracies, since a regional delay or even voxel-based delay, which considers the characteristics of smaller, albeit noisier, regions, might be more appropriate to capture the heterogeneity in tracer arrival times (16).

PET radiotracers are designed to target distinct biological functions, and thus their uptake, retention and clearance from tissue differ depending on the specific tissue they pass. In terms of compartmental modeling, [^15^O]H_2_O is described well by a one-tissue compartment model as it diffuses freely between blood and tissue, approximating an extraction fraction (EF) of one, while [^18^F]FDG is better described by a two-tissue model as it can enter into a metabolized state, and in addition, other tracers can also exhibit a number of other properties, which in turn alter the specific shape of the tissue TAC and the exact model used (17).

The conventional approach of delay correcting mean tissue TACs to an input function may therefore not be adequate due to both the noisy nature of single voxels with high time resolution as well as the different characteristics of radiotracers. In this article we explore a new method of estimating the delay of these tissue TACs, which involves fitting the cumulatively summed TAC to increase the robustness of the fit. Additionally, we examine the performance of this algorithm across a range of tracers, each with distinct noise characteristics.

To assess the performance, we will compare our proposed method (18) to two commonly applied methods, namely cross-correlation and a one-tissue compartment model including delay (19). Herein, we perform an evaluation on both synthetically produced TACs across a range of delays and levels of noise. Whole-body delay maps are calculated for each method for three tracers covering a large range of EFs.

## 2 Methods

### 2.1 Data acquisition

Dynamic PET scans from 15 patients were used in the development and testing of the algorithm, divided equally (*n* = 5) among each of the tracers: [^15^O]H_2_O, [^18^F]FDG and [^64^Cu]Cu-DOTATATE. The patients were scanned on a Siemens Biograph Vision Quadra PET/CT scanner with an axial coverage of 106 cm with a scan start approximately 10 s prior to tracer bolus injection. Data were reconstructed using a maximum ring difference (MRD) of 85 in a 440 × 440 voxels matrix resulting in a 1.65 mm in-plane voxel size. Raw list mode data were subsequently reconstructed using an ordered subset expectation maximum (OSEM, 4 it., 5 subsets) algorithm with scatter, point-spread function, time-of-flight and CT-based attenuation correction applied. The reconstruction filter used was a 3D Gaussian filter with 2.00 mm FWHM for [^15^O]H_2_O and an all-pass for [^18^F]FDG and [^64^Cu]Cu-DOTATATE. For [^15^O]H_2_O, the frame durations were 40 frames × 1 s + 5 frames × 4 s + 6 frames × 10 s + 3 frames × 20 s. For [^18^F]FDG and [^64^Cu]Cu-DOTATATE, frame durations were 40 frames × 1 s + 10 frames × 5 s + 15 frames × 10 s + 6 frames × 60 s. For analysis of TACs, the frame mid-times are used.

An image-derived input function (IDIF) was derived from a TotalSegmentator (20) segmented aorta subsequently transferred to the PET scan. The entire aorta was divided into four segments: the ascending part of the aorta, the aortic arch, the proximal descending part of the aorta and the distal descending part. The proximal descending part of the aorta was used to derive the IDIF across all tracers as the average time-activity curve within a 1 ml volume of interest, positioned lengthwise inside to avoid partial volume effects.

The choice of tracers included ones that are often used in dynamic PET imaging to measure blood flow and rate of metabolis, [^15^O]H_2_O and [^18^F]FDG, while [^64^Cu]Cu-DOTATATE was included to test the performance and limitations of the proposed model on a tracer with very low signal-to-noise ratio and first pass-extraction fraction.

The project was approved by the Departmental Review Board Rigshospitalet, University of Copenhagen on 17 September 2021. All patients provided written informed consent prior to inclusion.

### 2.2 Delay map algorithm

The algorithm estimates the onset times for the input function and a tissue time-activity curve independently and subtracts the two to estimate the delay between the input and the tissue ($\Delta T=T_{\mathrm{tissue}}-T_{\mathrm{input}}$). Both the input function and the tissue TAC go through the same processing as described below.

Due to the inherent noisy characteristics of single voxels, several approaches were employed to sensibly handle the noise. First the TAC was summed cumulatively. This eliminates most of the approximate Gaussian distributed noise. Secondly, plateaus were detected and removed in case of noise in the early part of the TAC before fitting the summed TAC for up to 10 different ranges to find the best matching fit within a set of criteria. In case of no acceptable fits, the average TAC of a 5 × 5 × 5 cube around the failed voxel was calculated and the algorithm was rerun on these average TACs only. This cube will have a volume of 0.56 ml for the [^15^O]H_2_O scans and 0.68 ml for the [^18^F]FDG and [^64^Cu]Cu-DOTATATE scans.

#### 2.2.1 Fitting the summed TAC

For ease of fitting, a simple fitting function with few parameters was proposed, which was empirically found to fit different types of TACs well:

(1) $$\begin{array}{c} A_{\mathrm{tissue}}(t;a,b,c)=\frac{a}{1+\exp\left(\frac{c-t}{b}\right)}, \end{array}$$

Where *a* is the amplitude or saturation level when *t* is large, i.e., t≫c; *b* indicates how rapidly the function transitions from 0 to the saturation level, *a*; and *c* is the center point around which the function transitions. Here b=0 means that the change happens instantly.

**Figure 1:**
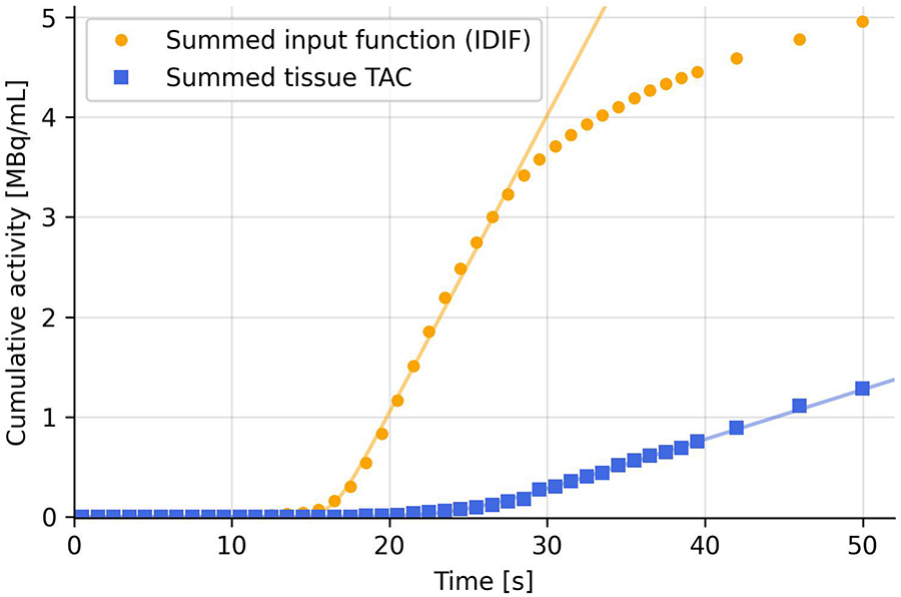
Summing the TACs cumulatively eliminates most of the noise and hence increases robustness of the fit using Equation (2).

This function fits the TACs from zero activity at the beginning and up to a saturation level. However, as observed in Supplementary Figure S1, time-activity curves for single voxels can be inherently dominated by noise, which in turn complicates the fitting process and the robustness of fitting directly to them. Thus, to reduce the effect of the noise and increase the robustness of the fit, the TACs are summed cumulatively, and fitted by the integral of $A_{\mathrm{tissue}}(t)$ (see Figure 1):

(2) $$A_{\mathrm{sum}}(t;a,b,c)=\int_{0}^{t}A_{\mathrm{tissue}}(t^{\prime })dt^{\prime }=ab\;\ln\left[\exp\left(\frac{c-t}{b}\right)+1\right]+a(t-c),$$

where *a*, *b* and *c* were defined in Equation (1). The onset time being determined from the fitting parameters as

(3) $$\begin{array}{c} T=c-3.5\cdot b. \end{array}$$

As *c* is the central point of transition from flat to a rising curve, the onset point lies before this time. To compensate, a correction term which depends on the tightness of the transition, *b*, is added. The constant value of 3.5 has been chosen empirically to provide a uniform appearance across the parameter maps depicted in Figures 3A,B.

While summing the TACs, the frame durations [the *dt'* in Equation (2)] must also be multiplied to each TAC data point to avoid abrupt changes at the points where the frame timing changes (see Supplementary Figure S2 for a constant activity curve sampled at varying time intervals with and without the correction). The summed TAC is thus:

(4) $$\begin{array}{rl} TAC_{\mathrm{sum}}(t) & =\overset{t}{\underset{i=0}{\sum }}(TAC(i)\cdot (\mathrm{FrameMidTime}(i+1)\\ & -\mathrm{FrameMidTime}(i))). \end{array}$$

#### 2.2.2 Simple plateau detection

**Figure 2:**
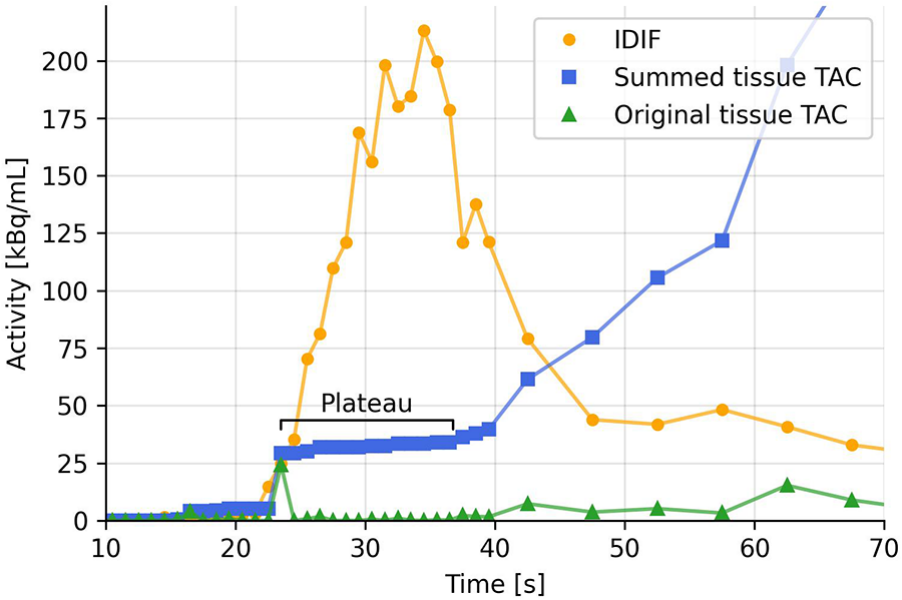
Example TAC from a voxel in the arm of injection (green), cumulatively summed TAC (blue), and IDIF (yellow). Possible partial volume effects in the early part of the TAC creates double spikes as the tracer passes tissue twice (venous pass and a later arterial pass). This creates plateaus in the summed TAC.

Figure 2 shows an example TAC with noise spikes, and how these can create steps and plateaus when the TAC is summed cumulatively. These steps bias the fitting function and might set the onset time at the first step, where the second step would be the arterial pass. To mitigate this, a simple plateau detection algorithm is utilized to detect when 3 or more data points lie within a band of 0.1% of the max value of the first two minutes of the summed TAC. The threshold is chosen to be relative to the TAC to accommodate TACs of different amplitudes and can be fine-tuned for an either more or less sensitive detection of plateaus. Only the first two minutes are checked, to prevent the tail of the TAC from being falsely detected as a plateau. When a plateau is detected, everything before that is set to zero and every following point is lowered by the value of the plateau.

#### 2.2.3 Parameter fitting ranges

The fitting function, Equation (2), tends to the straight-line asymptote: $A_{\mathrm{sum}}(t\;\gg \;c)\to a(t-c)$. However, the summed TACs usually fall off after a few minutes. To obtain the best possible fit to the upslope, up to 10 different ranges are fitted. The endpoints of the fitting ranges are selected based on when the summed TAC starts separating from the asymptote of the fitting equation. This is detected by computing where the absolute values of the second derivative of the summed TAC are highest. This way, both cases where either the summed TAC falls off below the fit asymptote or rises above it (for example due to a high uptake rate combined with a low clearance rate) are considered.

These points are identified through simple peak detection (21) by comparing adjacent points, and the top 10 peaks with the largest peak heights are chosen as the endpoints.

The fitting parameters were restricted to $0.1\frac{\mathrm{Bq}}{\mathrm{ml}\cdot s}\leq a\leq 2\times 10^{7}\frac{\mathrm{Bq}}{\mathrm{ml}\cdot s}$; 0.01s≤b≤10s; and 0s≤c≤60s, which were set broad enough to accommodate all types of summed TACs but more importantly to provide a starting point for the fitting function.

#### 2.2.4 Filtering the fits and choosing the best ones

After fitting one range of the summed TAC, as described above, the fit is tested against a set of criteria as follows. After checking for any fitting errors returned by the fitting function, the goodness of fit (GoF: $R^{2}$) is calculated and evaluated against a lower limit of 0.8. Next, the onset time from Equation (3) is calculated, which must be positive and lie before the endpoint of the fitting range (as found in the previous subsection).

The top half of the fits with the highest $R^{2}$ are collected and the median of their fitting parameters are calculated. From these, the onset time of the TAC is calculated using Equation (3).

### 2.3 Validation against other methods

The proposed method is compared against simple cross-correlation and a one-tissue compartment model with incorporated delay (19) to determine the strengths and weaknesses of each. For cross-correlation, the point of highest correlation is found, and for the one-tissue compartment model, an “endtime” of 180 s was used while all other parameters were kept at their default setting [as given in (19)].

### 2.4 Synthetic TACs

Synthetic TACs ($C_{T}(t)$) were generated by applying a one-tissue compartment model,

(5) $$\begin{array}{c} \frac{dC_{T}(t)}{dt}=K_{1}C_{A}(t)-k_{2}C_{T}(t), \end{array}$$

to an input function ($C_{A}(t)$) for a range of combinations of rate constants ($K_{1}$ and $k_{2}$), delays and added Gaussian distributed noise, as well as for input functions from different tracers. The three methods described above were tested on all the synthetic TACs to evaluate how well each method were able to estimate the known delay between the input function and the synthetic TAC.

Delay between tissue TAC and input function was added by moving the tissue TAC and interpolating to the original time sampling.

Noise was added to the tissue TAC by multiplying each timepoint by a random number from a Gaussian distribution centered at 1 with a standard deviation of σ. The standard deviation is the parameter used to vary the amount of noise. Any points with a negative value after noise was added were set to 0.

The ranges for each of the parameters used are given in Table 1.

**Table 1:** Ranges of parameters for synthetic tissue TACs.

	Start value	Step size	Stop value	Total
$K_{1}\left[\frac{\mathrm{ml}}{100\;g\cdot \min}\right]$	1	3.5	350	100
$k_{2}[\mathrm{mi}n^{-1}]$	0.05	0.03	3	100
Delay [s]	−10	1	10	21
Noise, σ [unitless]	0	0.1	0.5	6

The standard deviation range of the noise was chosen to match the noise level in the acquired PET data. A standard deviation of σ=0.1 approximately corresponds to the noise of a few hundred voxels averaged, while a standard deviation of σ=0.4 roughly corresponds to the noise of a single voxel. We will refer to these two noise levels as “low noise” and “high noise”.

## 3 Results

### 3.1 Synthetic TACs

**Figure 3:**
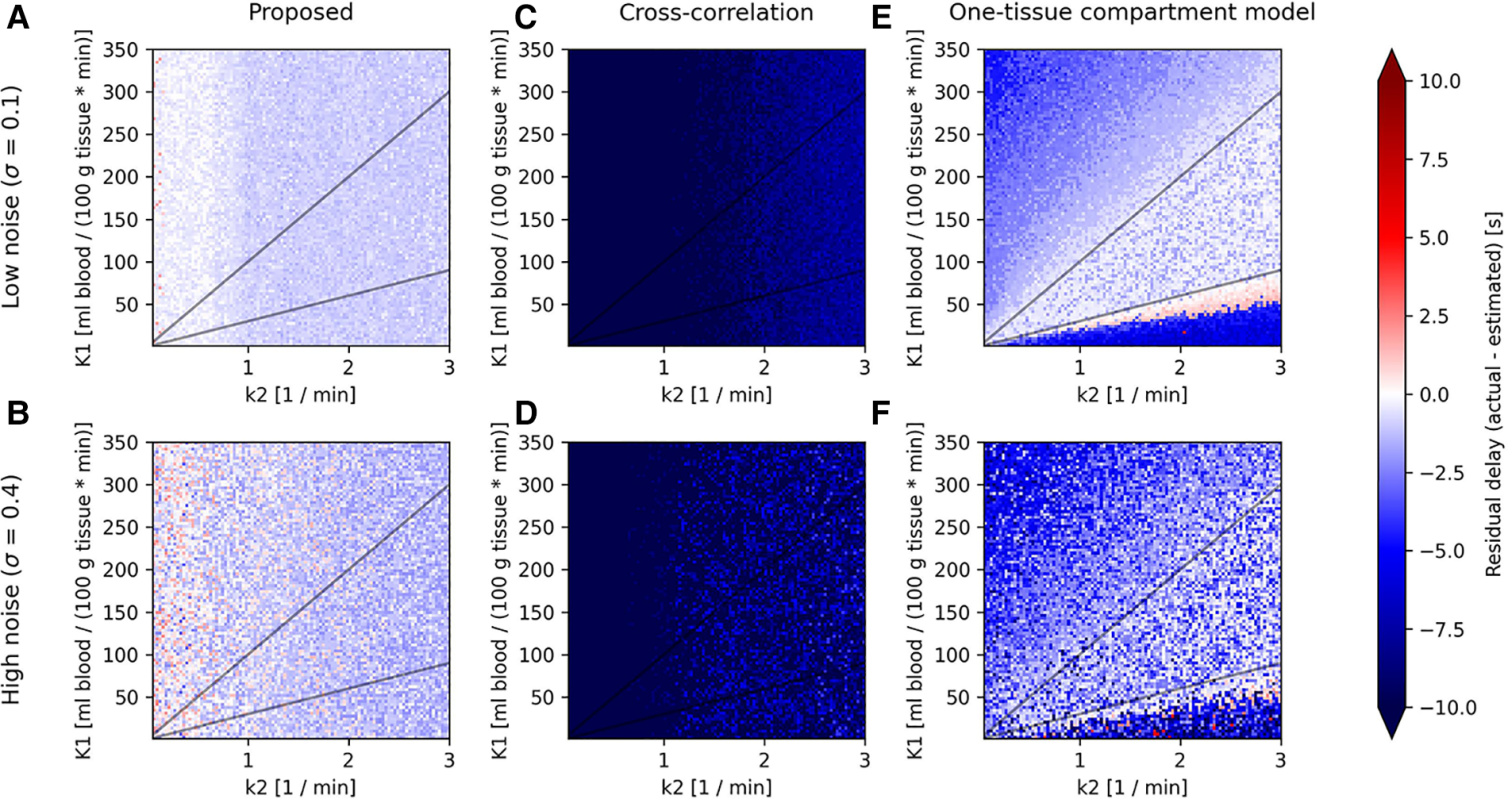
Parameter maps for the proposed model (left column), cross-correlation (middle column) and one-tissue compartment model (right column) showing the residual delay (difference between the actual delay and estimated delay) for synthetic TACs created from a one-tissue compartmental model using an IDIF of the tracer [^15^O]H_2_O as $C_{A}(t)$ in Equation (5), across combinations of $K_{1}$ (horizontal axis) and $k_{2}$ (vertical axis), linearly spaced in the range $1\frac{\mathrm{ml}}{100\;g\cdot \min}\leq K_{1}\leq 350\frac{\mathrm{ml}}{100\;g\cdot \min}$ and $0.05\;\mathrm{mi}n^{-1}\leq k_{2}\leq 3\;\mathrm{mi}n^{-1}$. Shown here for two levels of noise (low noise: **A**, **C**, and **E**; and high noise: **B**, **D**, and **F**) and for an actual delay between input function and tissue TAC of 0 s, as the parameter maps do not change significantly between different actual delays.

Figure 3 shows the residual delay between the known delay added to the tissue TAC and the delay estimated by each model. A negative residual delay indicates that the tissue TAC is estimated to be delayed to a later time than necessary. Each combination of $K_{1}$ and $k_{2}$ corresponds to a specific tissue TAC shape as given by Equation (4), where the left side of the parameter maps represent steadily increasing TACs with low rate of clearance and high uptake, while along the bottom the TACs have low uptake and a high rate of clearance, resulting in low signal TACs. Between these two extremes is the more physiologically relevant area with $30\frac{\mathrm{ml}}{100\;g}<\frac{K_{1}}{k_{2}}<100\frac{\mathrm{ml}}{100\;g}$.

All models show predominantly negative residual delays over the entire parameter map, indicating that the estimated delay was greater than the actual delay. Visually, the proposed model shows an overall consistent residual delay, cross-correlation shows very negative residuals, while the one-tissue compartment model with incorporated delay shows low residual delays in the middle of the parameter map for low noise, although as the noise increases, so does the residual delay. Both the proposed model and cross-correlation shows no dependency on $K_{1}$.

**Figure 4:**
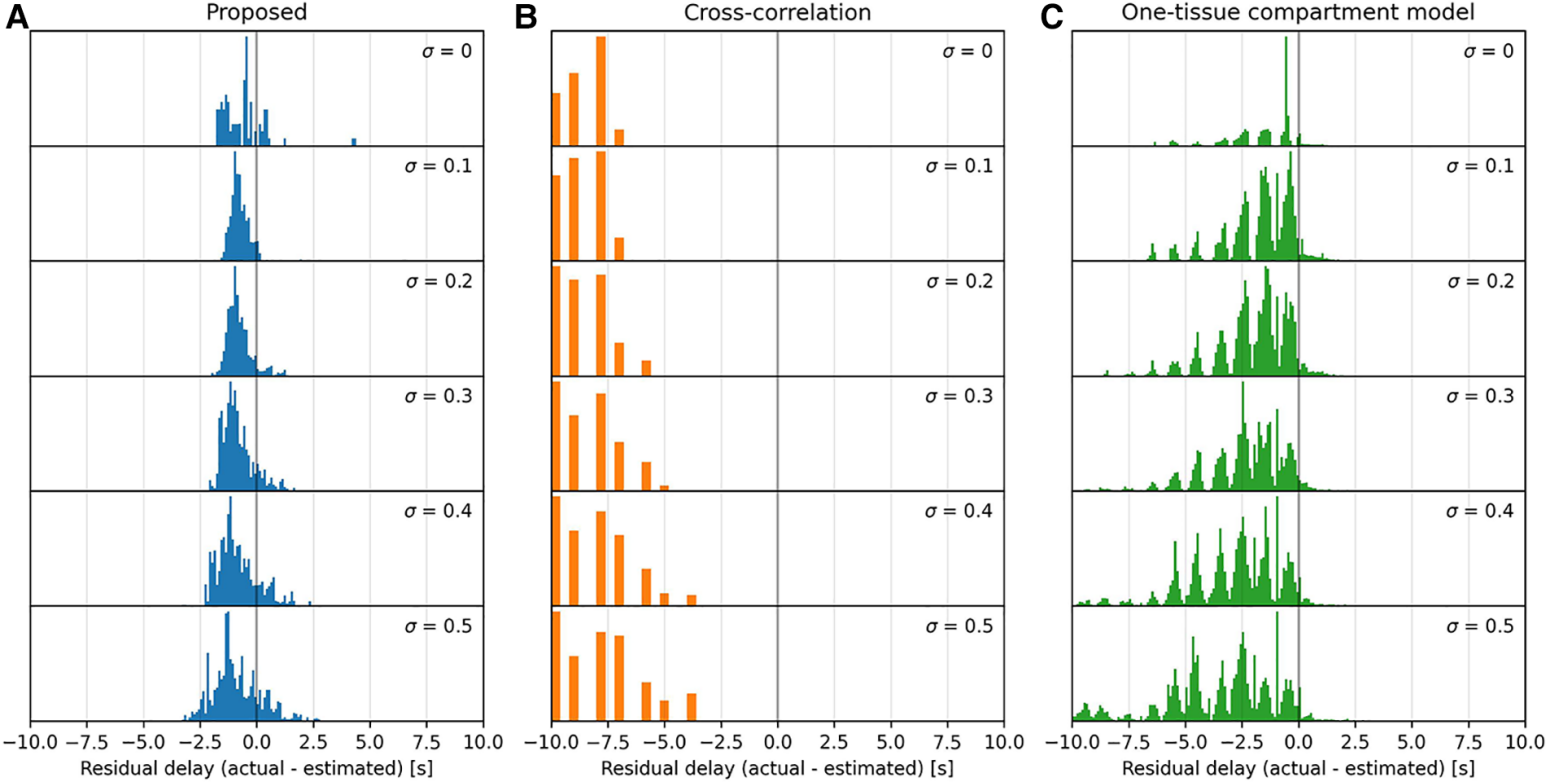
Histograms of the parameter maps in Figure 3 across a range of noise levels, 0≤σ≤0.5, downward, for the three models tested: proposed (**A**), cross-correlation (**B**), and one-tissue compartment model (**C**). Note that the *x*-axis is bounded in a range from −8 s–+8 s, and that the residual delay of cross-correlation mainly lies below this range.

Figure 4 shows the distributions of residual delays across the whole parameter map for increasing levels of noise.

The proposed model displays a moderately consistent residual delay of $-0.75\pm 0.38\frac{\mathrm{ml}}{100\;g\cdot \min}$ for low noise (σ=0.1) to $-0.85\pm 0.88\frac{\mathrm{ml}}{100\;g\cdot \min}$ for high noise (σ=0.4).

Cross-correlation displays a strong negative residual delay across the whole parameter map with mean values ranging from $-12\pm 6\frac{\mathrm{ml}}{100\;g\cdot \min}$ for low noise (σ=0.1) to $-13\pm 6\frac{\mathrm{ml}}{100\;g\cdot \min}$ for high noise (σ=0.4). The residual delay increases rapidly as $k_{2}$ decreases.

The one-tissue compartment model displays a wavy histogram with valleys at whole numbers and smaller distributions in between, peaking at half-integers. It has a mean of $-1.5\pm 1.5\frac{\mathrm{ml}}{100\;g\cdot \min}$ for low noise (σ=0.1) to $-2.9\pm 2.1\frac{\mathrm{ml}}{100\;g\cdot \min}$ for high noise (σ=0.4). The one-tissue compartment model appears to perform well in a triangular region (indicated by the diagonal lines in Figure 3) bounded by the distribution volume in the range $30\frac{\mathrm{ml}}{100\;g}<\frac{K_{1}}{k_{2}}<100\frac{\mathrm{ml}}{100\;g}$. However, as the noise increases this area also becomes increasingly noisy.

### 3.2 Whole-body delay maps

**Figure 5:**
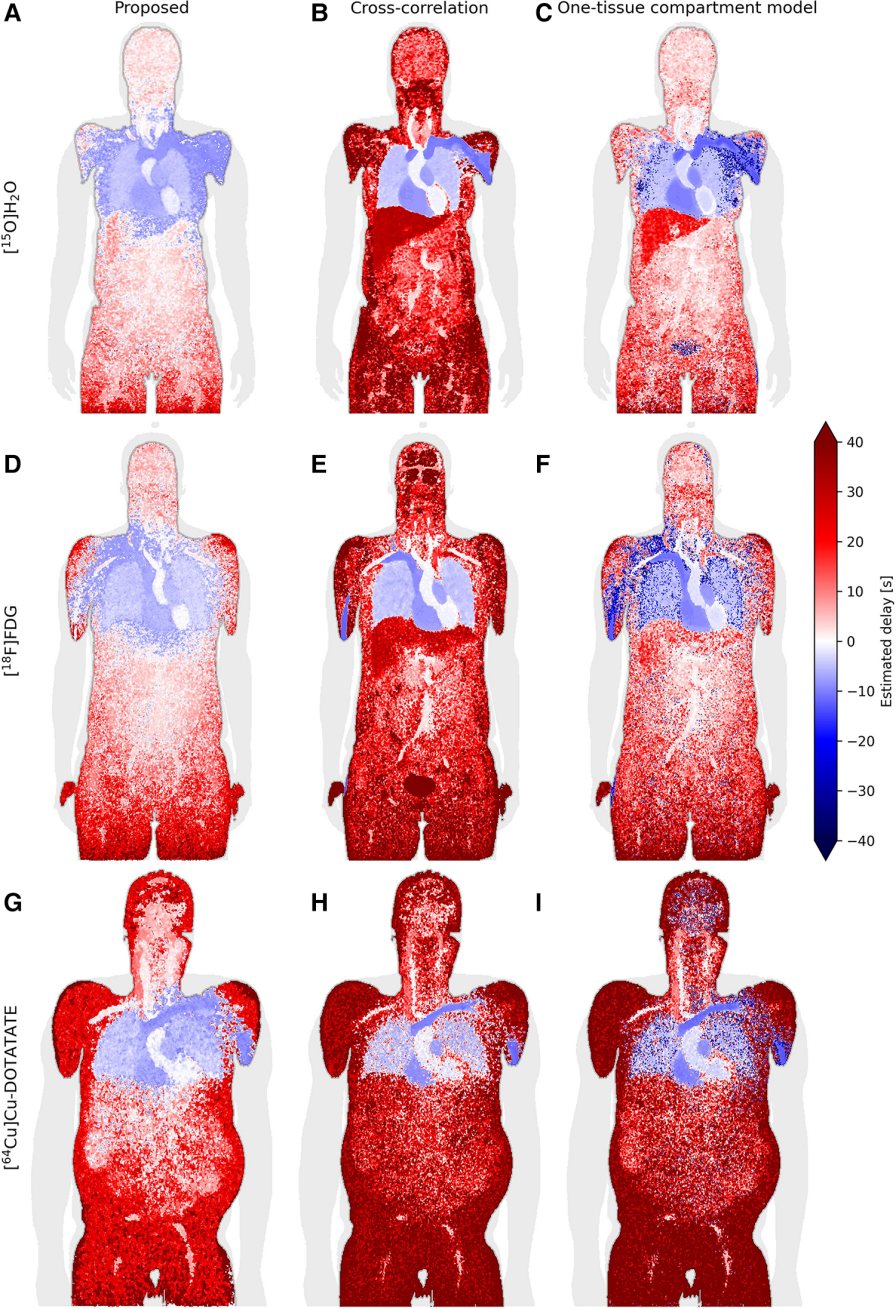
Coronal slice of whole-body delay maps for the proposed model (left column/subfigures **A**, **D**, and **G**), cross-correlation (middle column/subfigures **B**, **E**, and **H**) and a one-tissue compartment model with delay included (right column/subfigures **C**, **F**, and **I**), across the tracers [^15^O]H_2_O (top row), [^18^F]FDG (middle row) and [^64^Cu]Cu-DOTATATE (bottom row).

Figure 5 displays whole-body delay maps for the three models across three tracers. It is observed that [^15^O]H_2_O generally gives the least noisy delay maps, whereas [^64^Cu]Cu-DOTATATE gives the most noisy.

Generally, across all models and tracers, negative delays are observed in the vein of injection, heart and lungs as is expected with the input function derived from the descending part of the aorta. The proposed model additionally estimates negative delays in most of the arm of injection as well as the superior liver, while one-tissue compartment model estimates negative delays in the arm of injection with good separation of the liver. Cross-correlation displays very sharp separation of organs, for example between the lung and liver as well as between the vein of injection and the rest of the arm, however, it generally seems to overestimate the delay.

For the [^18^F]FDG delay maps, cross-correlation estimates a mean delay across the tested patients of 100 s ± 60 s in the brain, while the other models estimate a delay of around 4 s ± 4 s.

The one-tissue compartment model displays good separation between organs, particularly the lungs and liver for [^15^O]H_2_O. For [^18^F]FDG, this separation becomes less defined, while the delay in the aorta—the location where the IDIF is derived from—is estimated to be around −2 s ± 2.5 s. For [^64^Cu]Cu-DOTATATE, this model estimates spots of negative delays around the body and most noticeably in the brain.

### 3.3 Organ delay distributions

**Figure 6:**
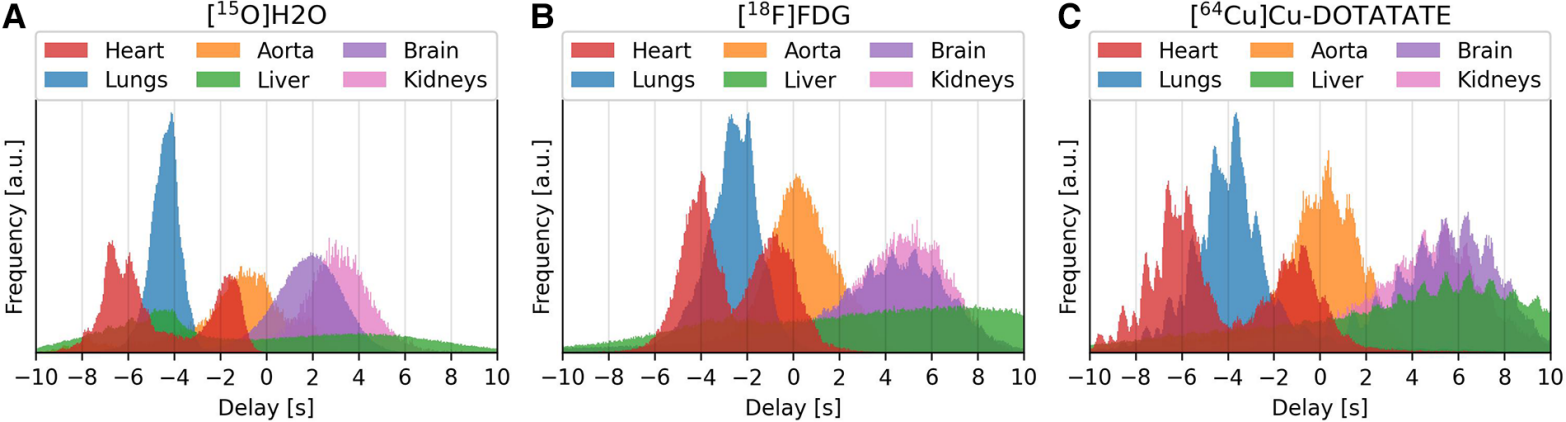
Delay distributions of selected organ for the tracers [^15^O]H_2_O (left/subfigure **A**), [^18^F]FDG (center/subfigure **B**) and [^64^Cu]Cu-DOTATATE (right/subfigure **C**).

Figure 6 shows the delay distributions of selected organs estimated by the proposed model across the three tracers. The organs have been segmented using TotalSegmentator (20).

Visually, the distributions for [^15^O]H_2_O are smooth, while for [^18^F]FDG and [^64^Cu]Cu-DOTATATE each organ distribution contains several smaller peaks. These smaller peaks occur at a rate corresponding to a frequency of approximately 1 Hz.

The distributions show the temporal progression of the tracer: the lungs can be observed to be located in between the right and left ventricle, after which the tracer enters the aorta and later reaches the other organs. The liver, however, spans the whole time scale, with the first half being the superior part of the liver.

### 3.4 Effect of voxel-wise delay correction on parametric images

**Figure 7:**
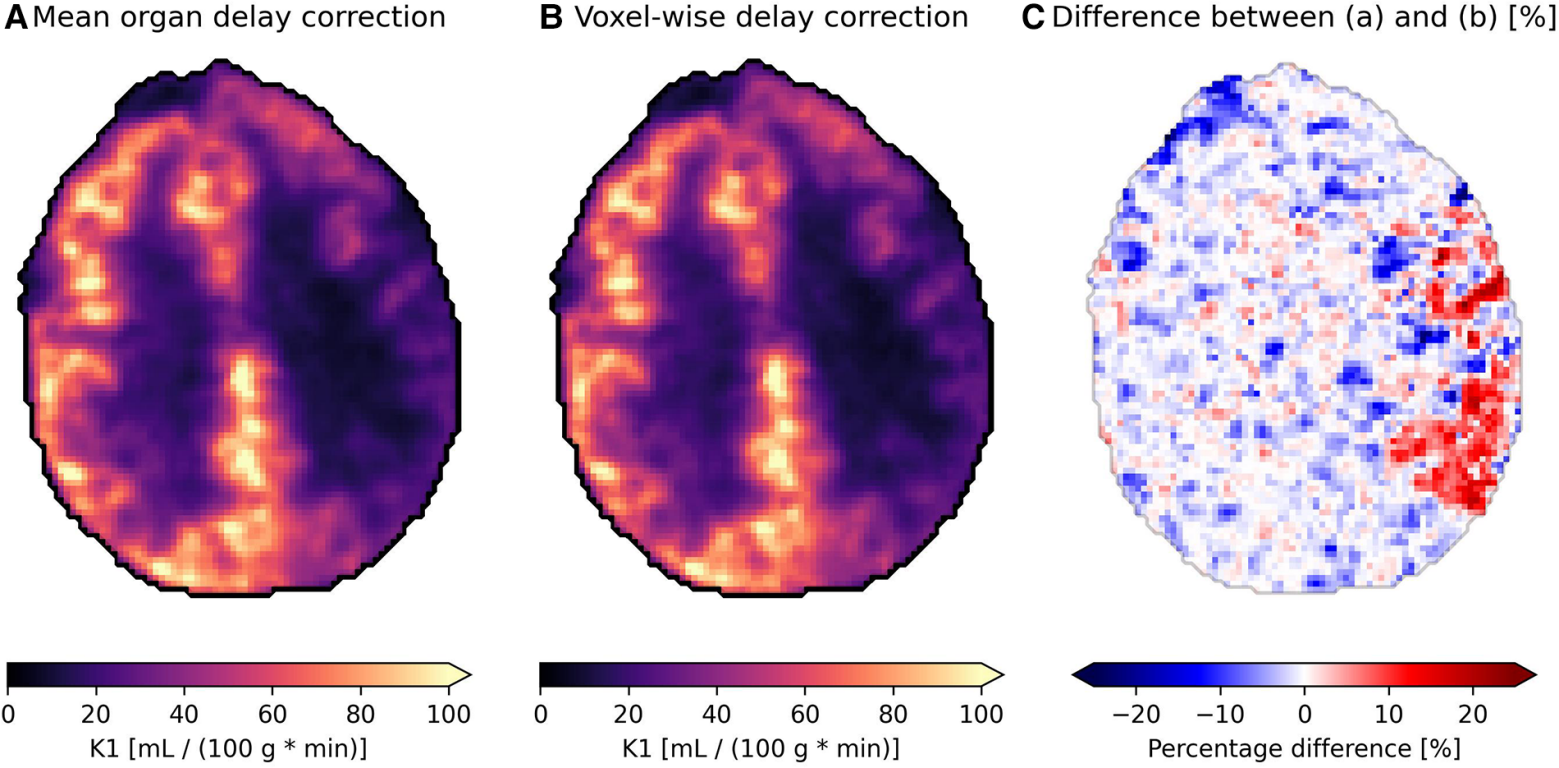
Parametric images showing the blood flow {*K*_1_, [ml blood/(100 g tissue * min)]} of an axial slice of brain in a patient with carotid stenosis on their left side (the right side in the images) receiving the tracer [^15^O]H_s_O. (**A**) is delay corrected using the mean organ time activity curve, while (**B**) uses voxel-wise delay correction with delay correction, and (**C**) shows the relative difference between the two.

The effect of delay correction can be observed from Figure 7, where subfigure (a) shows the modeled blood flow with a common delay correction applied to all voxels based on the mean time-activity curve for the organ (found using the proposed model), which is a common approach to adjust for delays in tracer arrival. Subfigure (b) presents a more refined approach, applying voxel-wise delay correction based on the proposed model. This accounts for the differences in tracer arrival time between voxels and on a regional basis. The relative difference between the two approaches is depicted in subfigure (c), where a difference of around 15%–25% can be observed on the side which is most affected by carotid stenosis.

## 4 Discussion

The negative delays observed in Figure 5 in the arm and shoulder in the tracer injection arm, as well as in the superior part of the liver for the proposed model, may be attributed to partial volume effects. A large amount of concentrated radiotracer was administered via an antecubital vein injection, which, as it travels to the heart through the venous system, affects the tissue surrounded by the high concentration bolus such as the rest of the arm and the shoulder. High levels of activity are also present in the heart, which, together with the heartbeat and breathing, can contribute to too-early estimates of the onset times in the superior part of the liver, and thus delay between it and the IDIF, as observed in the delay maps.

The above mentioned TACs can therefore exhibit “double-peaks”: the first for the venous bolus passage that affects it by partial volume or scatter-related effects and the second for the arterial pass. The desired peak we aim to detect here is the second one. These double-peaks in the TACs can partly be resolved by searching for plateaus in the summed TACs, as described in the methods section. If there are long periods with little to no signal between two peaks, for example in the case of the arm or superior liver, the second peak should be selected. The distinction between two peaks is, however, not always visible as they can start to blur together, and sometimes the first peak might be selected in these cases.

Plateau correction had to be performed for each tracer in different amounts across the body. For H_2_O, the main concern was in the arm of injection and other close-lying tissue where the initial tracer bolus would create a spike early in the time-activity curves that would not correspond to the first arterial pass. For FDG or DOTATATE, the main concern was the high amount of noise present.

As mentioned, in case the noise is too great to produce a good fit, the average of a 5 × 5 × 5 cube of voxels would be calculated around the failed voxels and used as a new TAC to run the algorithm on once more. In patients this had to be performed for on average 0.3% of voxels for the [^15^O]H_2_O scans, 0.03% for the [^18^F]FDG scans and 3% for the [^64^Cu]Cu-DOTATATE scans. This would usually be in the extremities of the patient, such as the legs, hands, or extremities in the lower torso. The highest number of failed voxels happened in the DOTATATE scans as expected, due to its low first-pass extraction and thus low signal-to-noise ratio, however the FDG scans have a lower error rate than the H_2_O scans, which could be due to the slightly slower kinetic profile of [^18^F]FDG over [^15^O]H_2_O together with the smaller mean free range of the $\beta^{+}$ from ^18^F compared to ^18^O, giving rise to less partial volume effects and an overall more even TAC.

The one-tissue compartment model with incorporated delay works by fitting a one-tissue compartment model to the tissue TAC with included delay. The tracer kinetics of [^15^O]H_2_O can likewise be described very well by a one-tissue compartment model and, as such, it is expected to perform well when estimating the delay for this particular tracer. This is especially true for high signal-to-noise ratio TACs such as mean organ TACs, but as the noise increases, this affects the model to a high degree, as can be observed in the parameter maps in Figure 3 and in the associated distributions in Figure 4, where a significant broadening in the delay distribution appears with increasing noise.

The proposed model, however, does not use compartmental modelling to fit the tissue delay but rather detects the uprising slope of the TAC and input function separately. In Figure 3A, this model can be observed to result in an overall even but small residual delay across the whole parameter map. The non-zero systematic residual delay is likely due to fitting the upslopes and calculating the onset times individually for the input function and the tissue TAC. Similar behavior has been observed before using straight line tangents to determine the upslope effectively giving an offset between the two curves, in cases of a theoretical delay of 0 s (22). The proposed model is similar, but incorporates a curve around the onset, which is used to correct for this inherent offset, and is the reason to why the fit parameter *b*, being the tightness of the curve, is present in the calculation of onset time in Equation (3).

Cross-correlation, by nature, strongly favors co-aligning the peaks of the tissue TAC and the input function. In the case where $k_{2}$ is small (left side of parameter maps in Figures 3C,D) the tracer is not efficiently cleared from the tissue and a tissue TAC will instead accumulate steadily. This effectively moves its peak towards the end, which leads cross-correlation to estimate a very large delay between it and the input function, resulting in a large negative residual delay. On the right side of the parameter maps where $k_{2}$ is large, the TAC will have a very defined peak. Here the curves are delay-corrected such that the peaks align, although the peak of the tissue TAC actually appears a few seconds later than the input function peak, resulting in the negative residual delay, however, slightly more positive than for small $k_{2}$, c.f. in Figures 3C,D.

In the whole-body delay maps (Figure 5), cross-correlation can be observed to frequently estimate larger delays than the other two models, while also exhibiting clearly defined organs. Both are due to peak-weighing of cross-correlation. For example, the brain in the [^18^F]FDG delay map (Figure 5E) has a much larger delay for this model than any of the other delay maps, due to the brain's high glucose metabolism causing large amounts of the tracer to accumulate over time, effectively moving the TAC peak near the end where the correlation between the two curves is highest. This high delay would significantly overestimate the kinetic parameters.

As expected due to the free diffusivity of water in tissue, the one-tissue compartment model with incorporated delay performs very well for [^15^O]H_2_O. Notably, it is difficult for the proposed model to reliably estimate the liver due to both partial volume effects and motion effects, however, the one-tissue compartment model estimates delays of about 15–20 s corresponding to the venous part of its blood supply, which is also the main component of its blood supply. Ideally the liver should be modeled in relation to a dual input function, which considers both the arterial and venous parts of its blood supplies. This might influence the estimated delays of the one-tissue compartment model as well as the proposed model more than cross-correlation as they are more sensitive to the shape of the TACs, whereas cross-correlation typically favors aligning the peaks.

Also for [^18^F]FDG, the one-tissue compartment model shows bias in delay values. Notably the aorta, where the input function is also derived from, has an average delay of −2 s. The discrepancy here could stem from the fact that FDG has a lower extraction fraction than H_2_O and very different kinetics that match best with a two-tissue compartment model, making it noisier on top of the noise at the single-voxel level.

[^64^Cu]Cu-DOTATATE has a very low extraction fraction, especially in the brain, which increases the noise of the tissue TACs significantly. The one-tissue compartment model shows very noisy characteristics in the brain and lungs. The proposed model performs better with a better discrimination of the aorta and lower delays in the surrounding tissues, similar to the other delay maps. However, DOTATATE exhibits large amounts of noise due to its very low extraction fraction leading to many different plateaus in the summed TAC, which is a challenge for the proposed model, especially in the peripheral tissues. Experimenting with the plateau parameters here may give better results in specific cases.

From the delay distributions of the tracers in Figure 6, the shape of the organ histograms can be observed to change slightly, going from [^15^O]H_2_O with an extraction fraction close to 1, through to [^18^F]FDG, and to [^64^Cu]Cu-DOTATATE with a very low extraction fraction. The first histogram is smoother, which relates to how water is easily diffusible from blood to tissue. Whereas for FDG and DOTATATE, the heartbeat can be observed in each organ's histogram. This is due to their lower extraction fractions, effectively resulting in a higher vascular weighing of the signal.

The proposed model also provides more Gaussian-distributed organ delays, compared to cross-correlation and the one-compartment model which exhibit troughs and valleys at integer delays. Features such as the heartbeat and the temporal progression of the tracer are also clearly visible.

The perfusion difference in flow in Figure 7C between a mean organ delay correction method and a voxel-wise delay correction method using the proposed model is shown in Figure 7C. It is apparent that tissue with longer delays, in cases such as carotid artery occlusion, can be underestimated by 15%–25%. Conversely, the kinetics in tissue with shorter delay times compared to the mean organ TAC delay are potentially being overestimated by a similar amount. In the case of Figure 7, the difference in delay between the affected region and healthy brain tissue was around 5 s ± 2 s.

The scope of this study did not include studying tumors; it was purely to test the performance of the proposed model and not the physiology. A more targeted and organ-specific model could be developed which takes into account the different types of tissue, as in the case of mixture models, where tumors are treated differently from other tissue types (23). However, the proposed model does not take any *a priori* decisions on the specific tissue type or the tracer used. It is thus independent of tissue type and tracer and should be broadly applicable. For all modeling approaches in general, delay correction is necessary when estimating kinetic parameters to avoid biased estimates.

In this study the tracer was administered as a bolus with an injection time being as fast as possible and was fully completed within the first 40 sec of scan time. Additionally, the injection was administered a few seconds after the scan started to allow for a few dead frames to be used as a baseline. The proposed model has therefore only been tested on bolus injections without considering variations in injection time.

## 5 Conclusion

We have developed a model capable of estimating the tracer arrival times in various tissues and organs across different tracers exhibiting different properties, such as their extraction fractions, half-life and kinetics. It is important to note that this model was only tested on data from the Siemens Quadra whole-body PET/CT scanner with high-temporal resolution but can, in principle, be extended to lower time resolution framing images with appropriate validation. Nonetheless, it performs well for a large range of noise levels and for a wide range of tissue TAC shapes as tested using the synthetically produced one-compartment TACs.

It performs better in high-noise environments and for low extraction fraction tracers, such as [^18^F]FDG, than the one-tissue compartment model with incorporated delay. This in turn makes it good as a single-voxel delay map and can easily be parallelized for shorter calculation times.

Additionally, the organ delay distributions are normally distributed, which the two other models tested here do not produce, and clearly show temporal features such as the heartbeat and the tracer progression through the body and organs.

## Data Availability

The data analyzed in this study is subject to the following licenses/restrictions: Patient confidentiality. Requests to access these datasets should be directed to thomas.lund.andersen@regionh.dk.
