# Supplementary material for: Single-voxel delay map from long-axial field-of-view PET scans
Source: Front Nucl Med. 2024 Apr 19;4:1360326. doi: 10.3389/fnume.2024.1360326 (PMC11440851; doi:10.3389/fnume.2024.1360326)

# Supplementary

Figure 1 – Input function and single-voxel tissue time activity curve (TAC) showing the standard noise levels.


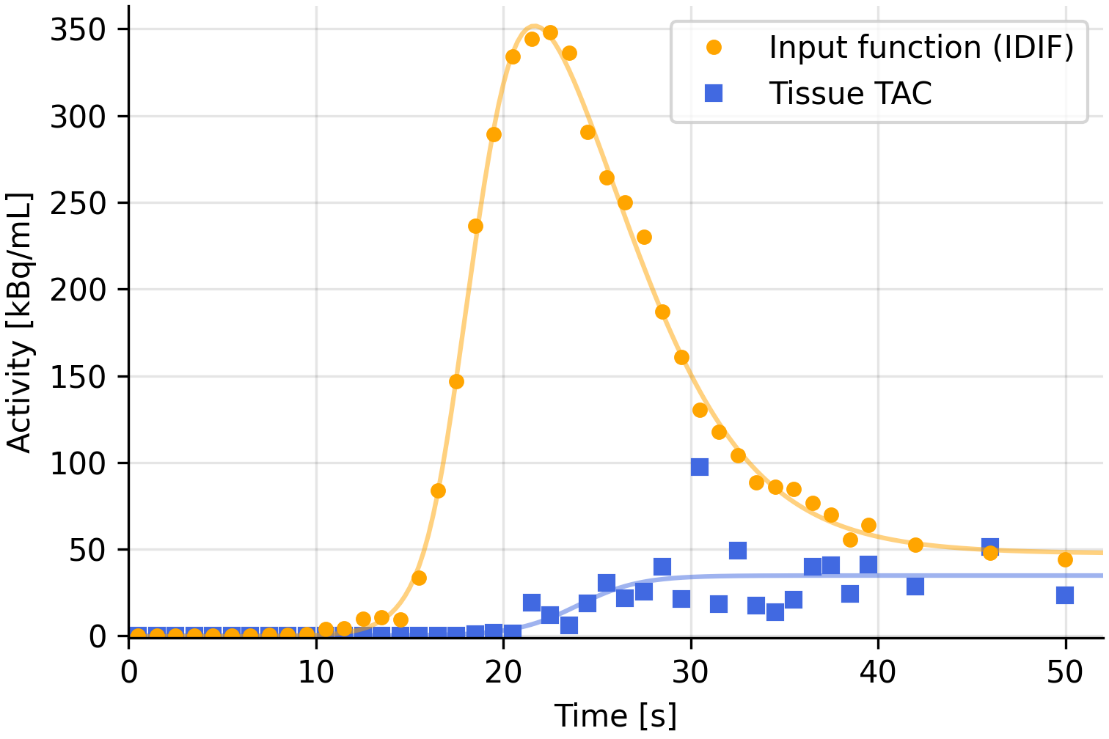


Figure 2 – Correcting the summed time activity curves for varying frame durations.


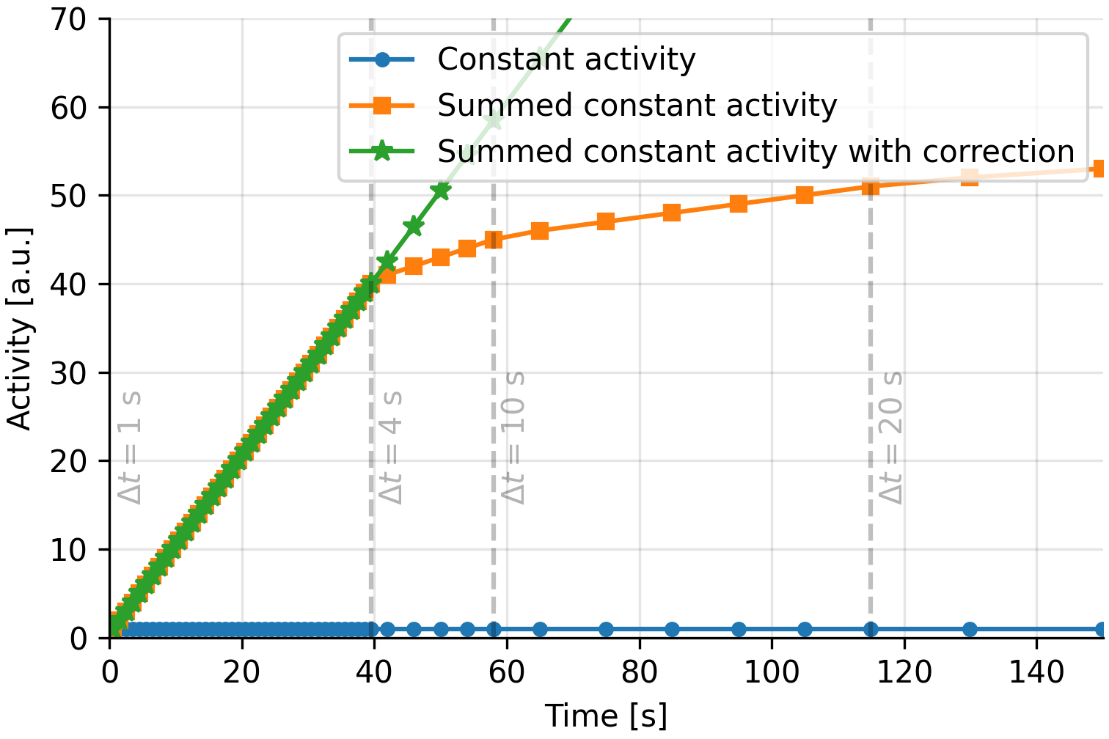

Supplement: Supplementary file 1 [file Datasheet1.docx]
